# Haemodynamic flow conditions at the initiation of high-shear platelet aggregation: a combined *in vitro* and cellular *in silico* study

**DOI:** 10.1098/rsfs.2019.0126

**Published:** 2020-12-11

**Authors:** B. J. M. van Rooij, G. Závodszky, A. G. Hoekstra, D. N. Ku

**Affiliations:** 1 Computational Science Lab, Informatics Institute, University of Amsterdam, Amsterdam, The Netherlands; 2 Mechanical Engineering, Georgia Institute of Technology, Atlanta, GA, USA

**Keywords:** high-shear thrombosis, microfluidic devices, platelet-rich plasma, porcine blood, von Willebrand factor, cell-depleted layer

## Abstract

The influence of the flow environment on platelet aggregation is not fully understood in high-shear thrombosis. The objective of this study is to investigate the role of a high shear rate in initial platelet aggregation. The haemodynamic conditions in a microfluidic device are studied using cell-based blood flow simulations. The results are compared with *in vitro* platelet aggregation experiments performed with porcine whole blood (WB) and platelet-rich-plasma (PRP). We studied whether the cell-depleted layer in combination with high shear and high platelet flux can account for the distribution of platelet aggregates. High platelet fluxes at the wall were found *in silico*. In WB, the platelet flux was about twice as high as in PRP. Additionally, initial platelet aggregation and occlusion were observed *in vitro* in the stenotic region. In PRP, the position of the occlusive thrombus was located more downstream than in WB. Furthermore, the shear rates and stresses in cell-based and continuum simulations were studied. We found that a continuum simulation is a good approximation for PRP. For WB, it cannot predict the correct values near the wall.

## 1. Introduction

One out of four deaths worldwide is somehow related to thrombosis [1]. In recent decades, researchers have shown an increased interest in high-shear thrombosis. Examples of high-shear thrombosis are a myocardial infarction or stroke due to atherothrombosis or an embolus. In particular, the behaviour of the protein von Willebrand factor (vWF) has received the most attention. The working of this protein is shear-dependent and, together with platelets, it is one of three pillars required for high-shear thrombus formation, next to a thrombogenic surface and high shear [2,3]. The exact role of high shear and its influence on the uncoiling of vWF is still under debate. A number of researchers have reported that a high-shear micro-gradient [4,5] correlates with platelet aggregation. Others state that a high shear itself is sufficient to form an occluding thrombus [6]. A third group found that the elongational rate is important [7]. Colace & Diamond [8] found that elongational flow is not necessary for vWF fibre formation on a collagen surface at extreme shear rates of 30 000 s^−1^. However, at lower shear rates elongational flow could play a role [9].

To study platelet aggregation under high shear, many researchers perform *in vitro* experiments using microfluidic devices. A diverse range of flow chambers are used that vary in geometry, coating, flow conditions, etc. Flow chambers coated with collagen type I or III and/or with vWF are mostly used. These coatings trigger platelet aggregation using platelet receptors that bind directly to collagen (GPVI and *α*_2_*β*_1_) and platelet receptors that bind to vWF (GPVI and *α*_2_*β*_1_) [10]. The differences in geometry determine the haemodynamic parameters, such as the shear rate, shear stress and their gradients. However, it is difficult to obtain the exact values of shear rates and stresses in these devices. Previously, we found that shear rates are underestimated and shear stresses overestimated in continuum simulations assuming blood as a Newtonian fluid with a constant viscosity [11]. Since most researchers [4,5,12,13] used this assumption, there is a clear need to investigate the details of the flow domain and flow conditions in the microfluidic experiments in high-shear thrombosis.

The objective of this study is to further investigate the role of high shear in microfluidic devices. In particular, we study the haemodynamic environment using a cell-based blood flow simulation in a newly designed microfluidic device [14]. The simulation results are compared with the initial thrombus formation in a microfluidic device. Our microfluidic device contains a stenotic area in which the shear rates are high ($\dot{\gamma }>3000\;{\text{s}}^{-1}$). It is designed in such manner that the flow conditions in the device can be analysed with cell-based simulations. Therefore, this study provides an example of how haemodynamic parameters could be investigated in the future.

Previously, we have shown that a cell-depleted layer in combination with high shear and a high platelet density might be a possible indicator for high-shear thrombosis [11]. In this study, we compare the cell-free layer from numerical transport modelling with experimental results on initial platelet aggregation. To test whether the cell-depleted layer in combination with high shear and high platelet flux can account for the distribution of platelet aggregates, we performed experiments with whole blood (WB) and platelet-rich plasma (PRP). With WB, a cell-free layer is present at the walls of the flow chamber, whereas PRP eliminates the cell-free layer throughout the whole domain owing to the lack of red blood cells (RBCs). We expect that the platelet flux is higher at the walls in WB than in PRP because of platelet margination. This might result in a differently sized and/or located platelet aggregate.

In the experiments, platelet adhesion and platelet aggregation occur on the coated type I collagen. The focus in these experiments is on (initial) platelet aggregation, because platelet adhesion is difficult to measure.

## 2. Material and methods

### 2.1. *In vitro* flow chamber experiments

#### 2.1.1. Microfluidic device design

**Figure 1.**
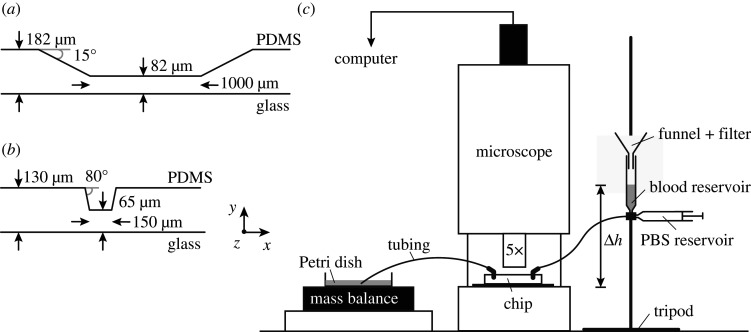
Side view of the geometric design of both microfluidic flow chambers: (*a*) Casa’s flow chamber and (*b*) van Rooij’s flow chamber. The depth of both flow chambers is 480 μm (*z*-direction, not shown). (*c*) A schematic of the experimental set-up. PBS, phosphate-buffered saline.

A new microfluidic device was designed to study platelet aggregation in a high-shear environment. This device contains eight flow channels with a length of 15 mm, width of 480 μm and height of 130 μm. Each channel contains a 50% stenosis with an 80° contraction and expansion angle and has a stenotic length of 150 μm (figure 1*a*,*b*). A mould for the newly designed flow chamber, called van Rooij’s flow chamber, was manufactured from brass using micro-machining (OM-1A 3-axis CNC machine; Haas Automation, Oxnard, CA, USA) based on a three-dimensional (3d) CAD model (SolidWorks, USA). A 0.127 mm square end mill was used to create the stenotic part in each channel. The micro-machining had an accuracy of approximately 25 μm, because this was the step size for alignment of the end mill to the brass mould. We aimed for a channel height of 140 μm with a 50% stenosis; however, after measuring the height we found an average stenotic height of 65.7 μm and an average channel height of 131 μm using a 3D laser confocal microscope (LEXT OLS400; Olympus). Images of the mould, images of the microfluidic device and height measurements of two flow channels are presented in the electronic supplementary material.

The devices were manufactured by pouring polydimethylsiloxane (PDMS) into the mould and allowing it to harden for 3 h at 65°C in an oven (Thermo Fisher Scienfic Inc., USA). After removing the PDMS device from the mould, the inlets and outlets of each flow chamber were created using a needle. This needle had the same size as the needles used to connect the tubing to the flow chamber. The last manufacturing step was surface plasma bonding of the PDMS part to a microscopic glass plate (20 × 75 mm) using a plasma cleaner (Harrick Plasma, New York, NY, USA). For a more detailed description of the microfluidic device manufacturing, see [15].

#### 2.1.2. Whole blood and platelet-rich plasma

Whole porcine blood was heparinized (3.5 USP units/ml) directly after collection (Holifield Farms, Covington, USA). PRP was created by sedimentation of the RBCs. WB was poured into a beaker and after 2 h the PRP layer was transferred to 50 ml tubes using a pipette. The platelet concentration of the PRP was counted using a haemocytometer (DHC-N01; Incyto, Republic of Korea) and was on average $4.17\times 10^{5}\;\text{platelets}/\mu \text{l}$. The normal range of the platelet concentration in a pig is $3.8\times 10^{5}-6.5\times 10^{5}\;\text{platelets}/\mu \text{l}$. The haematocrit of porcine blood is 38±2.1% [16]. This value was adapted from the literature, because the variation in the haematocrit of normal pigs is small. The experiments were carried out at room temperature within 8 h, starting from the moment the blood was collected.

#### 2.1.3. Test platform

Platelet aggregation was studied in a microfluidic device designed by Casa *et al.* [17] and in van Rooij’s microfluidic device as introduced above. The geometries of the flow chambers on each device are shown in figure 1*a*,*b*. An overview of the experimental set-up is shown in figure 1*c*. The flow chambers were coated with 0.1% collagen type I (Chrono-log Corp., Havertown, PA, USA) overnight at room temperature in a moist environment. Prior to the experiment, the flow chamber was rinsed with phosphate-buffered saline (PBS; saline 0.9%). During the experiment, the flow through the channel was regulated by a constant hydrostatic pressure (Δ*h*). This pressure head was controlled by an open syringe attached to a tripod at a certain height. The pressure heads were uniform for WB and PRP. The device was placed on the stage of a bright-field microscope (DM6000B; Leica Microsystems, Germany) with 5× magnification. Via tubing, the flow chamber was connected to the open syringe in which the blood was kept at a constant level to maintain the constant pressure head. The blood was filtered prior to the experiment using a funnel covered with 30 μm filter paper. Additionally, a syringe filled with PBS was connected to the valve where the open syringe was attached. The PBS was used to rinse the flow chamber and remove all air from the channel prior to the experiments. The same type of tubing was used to connect the outflow side of the flow chamber to a Petri dish. The outflow of blood was measured by a mass balance (Scout; Ohaus, Switzerland) holding the Petri dish. The evaporation of the blood mixture was measured by recording the mass of a Petri dish with a constant amount of a similar mixture of blood and water that was placed on an adjacent mass balance. The mass balance data were visualized and saved using LabView (National Instruments Corp., Austin, TX, USA). The clotting process was observed using the microscope, and images were acquired twice a second using a high-resolution CCD camera (Pixelfly; PCO, Kellheim, Germany).

The experiments were performed at different hydrostatic pressures. In table 1, the height and corresponding flow rates for both flow chambers are given. These flow rates are derived from the mass balance data. The flow rate is given in the SI unit m^3^ s^−1^ and can be converted to the unit μl s^1^ by multiplying the flow rate by 10^9^. In this study, the PRP experiments were compared with WB experiments at the same flow rate.

**Table 1.** The flow rates at multiple height differences.

chip design	height difference Δ*h* (mm)	flow rate WB (m^3^ s^−1^)	flow rate PRP (m^3^ s^−1^)
van Rooij	55	1.4 × 10^−9^	2.4 × 10^−9^
	115	2.4 × 10^−9^	4.4 × 10^−9^
	170	3.3 × 10^−9^	—
Casa	70	2.1 × 10^−9^	4.9 × 10^−9^
	100	3.0 × 10^−9^	6.8 × 10^−9^
	160	5.7 × 10^−9^	—

#### 2.1.4. Data analysis of the experimental results

**Figure 2.**
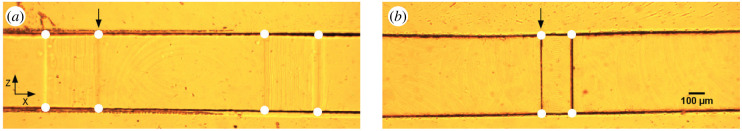
Microscopic images at the start of the experiment: (*a*) Casa’s flow chamber and (*b*) van Rooij’s flow chamber. The white points indicate the corners of the stenotic region. The black arrows point to the vertical line on which the alignment of the normalized pixel intensity was based.

Microscopic images acquired during the experiments were post-processed to obtain more information about the location of the occluded blood clot. In this analysis, we used the data from all our experiments performed with WB and PRP and with both flow chambers. The pixel intensities of grey-scale images were used to investigate the location of the platelet aggregates at occlusion. The experimental data of the different flow rates were used as one group, because the flow rates had no significant visible influence on the investigated intensity at occlusion. Note that in the WB experiments the platelet aggregates became visible because of an increase in intensity and in the PRP experiments because of a decrease in intensity. First, for both flow chambers, the start and end of the contraction and expansion areas were obtained in the 10th frame of the experiment. A semi-automatic algorithm was used in which the user needs to click on the eight corners of the microfluidic channel (or four corners in case of the van Rooij flow chamber) (figure 2). Next, the occlusion frame was determined from the mass balance data as the point in time when the mass reached its maximum. In multiple experiments, the mass plateaued at its highest level for a couple of seconds; in that case, the centre of the area was taken as the occlusion time. The corresponding occlusion frame was turned into a grey-scale image. The average pixel intensity of the grey-scale image was determined by averaging over the width of the flow chamber (*z*-direction) to find the occlusion location in the direction of flow (*x*-direction). All average pixel intensities were plotted in one figure. This was established by alignment of the right border of the contraction area (black arrows in figure 2). For the PRP data a lower averaged pixel intensity implied more platelets, whereas for the WB data a higher averaged pixel intensity was required. Therefore, the PRP data were inverted, so that they could be compared with the WB data.

### 2.2. Cell-based simulations

Cell-based blood flow simulations were performed to gain more insight into the transport physics of cells at the starting point of platelet aggregation. The *in vitro* experiments performed with van Rooij’s flow chamber are simulated with HemoCell (*Q* = 2.4 × 10^−9^ m^3^ s^−1^), a cell-based blood flow model validated for human blood [14]. We assumed that the porcine blood is a good model for human blood in terms of thrombosis. Therefore, the cell-based simulations of human blood are a good approximation of the blood flow in the microfluidic device (see Discussion for more details). Only a part of the flow chamber of 200 μm in front and 200 μm behind the stenotic section was modelled to reduce the computational time. The width of the channel (*z*-direction) was much larger than the height (*y*-direction) of the flow chamber (*w* ≫ *h*). Therefore, we could use a width of 50 μm instead of 480 μm in combination with a periodic boundary condition in the *z*-direction (figure 1), which further reduced computational time. The total domain size of the simulations was 572 μm × 130 μm × 50 μm. Note that the size of the channel influences the velocity profile, which affects the margination behaviour of the platelets. The cell-resolved simulation for WB had a tube haematocrit of 33% and contained 10 703 RBCs and 952 platelets and the simulation for PRP contained 941 platelets. The haematocrit was chosen to be lower than the average pig haematocrit of 38% to save computational time. The margination of platelets would not be affected by this slightly lower haematocrit. For both simulations, the plasma density was 1025 kg m^−3^ and the viscosity of the plasma was 1.1 × 10^−6^ m^2^ s^−1^. The blood flow was driven by a body force, and periodic boundary conditions in the direction of flow were used. The flow rate *Q* was matched to the experimental flow rate derived from the mass balance data. Therefore, the simulations had approximately the same flow rates as the experiments, *Q*_WB_ = 2.1 × 10^−9^ m^3^ s^−1^ and *Q*_PRP_ = 2.3 × 10^−9^ m^3^ s^−1^. The flow Reynolds number of the cellular simulation for WB and PRP was approximately 4 and the particle Reynolds number had a value of approximately 0.06. Both were calculated using the plasma viscosity. A time step of 5 × 10^−8^ seconds and a lattice size of 5 × 10^−7^ μm were used in cell-based simulations. The cell-resolved WB simulation ran on 600 cores on the supercomputer Cartesius (Surfsara, Amsterdam, The Netherlands) for 10 days and the PRP simulation ran on 256 cores for 5 days on the supercomputer Lisa (Surfsara, Amsterdam, The Netherlands). A physical time of 0.35 s was simulated for both WB and PRP. We did not simulate Casa’s flow chamber with HemoCell owing to its size. This would cost many more CPU hours. In Azizi *et al.* [18] and Alowayyed *et al.* [19] more details can be found about the computational performance of HemoCell.

Additionally, a continuum blood flow simulation of blood (with *ρ* = 1060 kg m^−3^ and *ν* = 3.3 × 10^−6^ m^2^ s^−1^) and PRP (with *ρ* = 1025 kg m^−3^ and *ν* = 1.1 × 10^−6^ m^2^ s^−1^) was performed. In those simulations, the fluid was assumed to be Newtonian. The shear rates and shear stresses obtained from the continuum simulations were compared with those obtained from the cell-based simulations.

#### 2.2.1. Data analysis of the simulation results

The shear rates and shear stresses in the *in silico* experiments were measured 1 μm from the glass plate wall (straight side) and the PDMS wall (stenotic side) for both cell-based and continuum simulations. The *xy*-component of the shear rate and stress were both derived from the flow field, because in this geometry the other components are negligibly smaller in comparison. The platelet count per second and platelet residence times were derived as described in [11]. The platelet count per second or platelet flux is the average number of platelets that were present on a certain location in the domain measured every 100 000 time steps divided by the total time of all time steps. The residence time of a platelet was defined as the time it takes a platelet to translocate its own diameter (2 μm).

## 3. Results

### 3.1. Cell-based flow simulations

**Figure 3.**
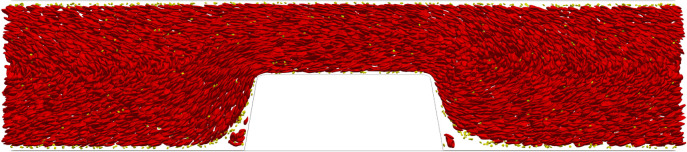
A side view of the cell-based WB simulation with *Q* = 2.1 × 10^−9^ m^3^ s^−1^ in van Rooij’s flow chamber at the last time step (*t* = 0.35 s). The RBCs are shown in red and the platelets yellow.

**Figure 4.**
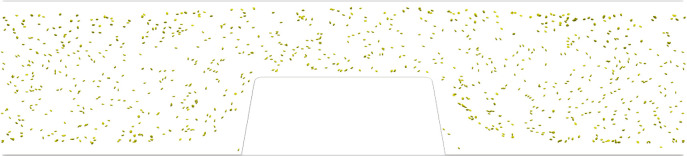
A side view of the cell-based PRP simulation with *Q* = 2.3 × 10^−9^ m^3^ s^−1^ in van Rooij’s flow chamber at the last time step (*t* = 0.35 s). Platelets are shown in yellow.

Figure 3 shows a side view of the WB cell-based simulation in van Rooij’s flow chamber at *t* = 0.35 s. The platelets (yellow) were marginated to the top and bottom walls of the flow chamber. Another interesting observation is that re-circulation areas were present in front of and behind the stenotic section. In those areas RBCs and platelets could be trapped. In figure 4, a side view is shown of the PRP cell-based simulation at *t* = 0.35 s. The platelets were present throughout the whole domain.

#### 3.1.1. Platelet density and platelet residence time in van Rooij’s flow chamber

**Figure 5.**
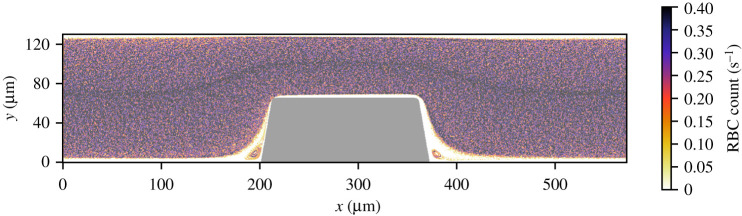
The RBC flux averaged over 0.30 s in van Rooij’s flow chamber for the cell-based WB simulation with *Q* = 2.1 × 10^−9^ m^3^ s^−1^.

**Figure 6.**
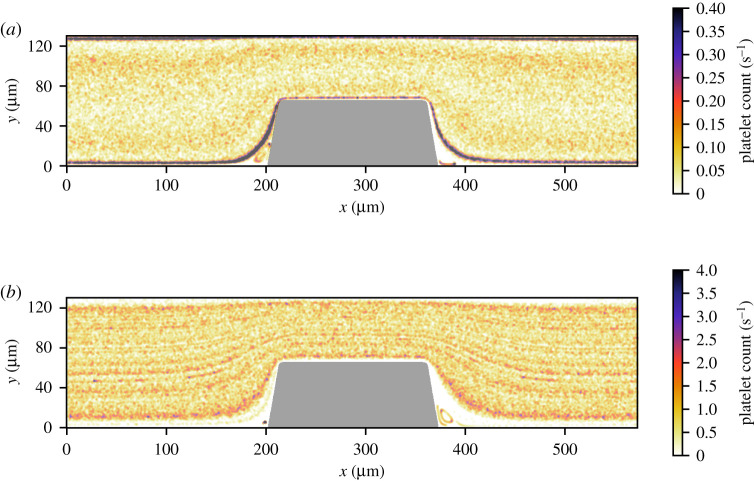
The platelet flux averaged over 0.30 s in van Rooij’s flow chamber for (*a*) the cell-based WB simulation with (*Q* = 2.1 × 10^−9^ m^3^ s^−1^) and (*b*) the cell-based PRP simulation (*Q* = 2.3 × 10^−9^ m^3^ s^−1^).

**Figure 7.**
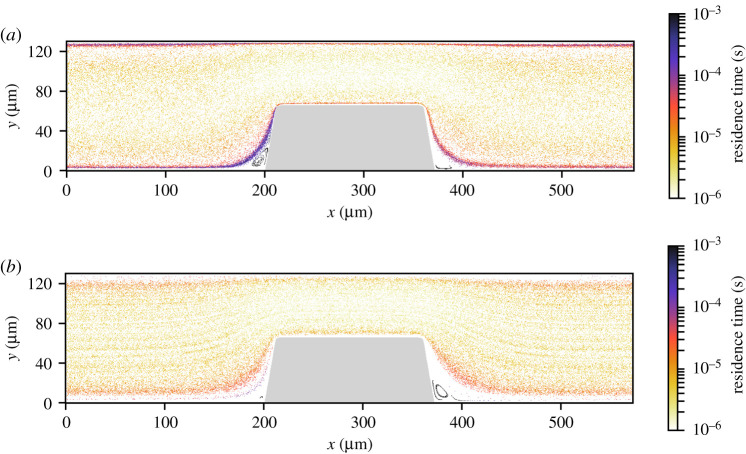
The platelet residence time averaged over 0.30 s in a flow chamber on van Rooij’s chip for (*a*) the cell-based WB simulation (*Q* = 2.1 × 10^−9^ m^3^ s^−1^) and (*b*) the cell-based PRP simulation (*Q* = 2.3 × 10^−9^ m^3^ s^−1^).

The RBC count per second, platelet count per second and the platelet residence times were obtained from the cell-based WB and PRP simulation. Figure 5 shows the RBC flux averaged in the *z*-direction of the geometry. There was a high flux of RBCs in the middle of the flow chamber and a lower flux at the walls of the flow chamber. Additionally, the RBC-depleted layer was clearly visible and had an average thickness of 5 μm. The cell-depleted layer was defined as the layer that contains only 1% of the volume fraction of RBCs. The width of the cell-depleted layer was not constant because of the geometry of the flow chamber. The platelet counts per second, or platelet flux, for WB and PRP are shown in figure 6. In this figure, it is clearly visible that, for the WB simulation, the highest flux of platelets was located in the RBC-depleted layer next to the flow chamber’s wall. Additionally, a slightly higher platelet flux could be observed at one-quarter (*y* = 30 μm) and three-quarters (*y* = 100 μm) of the channel height. At the top and bottom walls of the flow chamber, we observed a platelet-free layer. This is clearly visible in figure 3. This platelet-free layer was larger at the expansion area than at the contraction area. In the cell-based PRP simulation, we observed a larger platelet-free layer than in the WB simulation. The platelet flux near the wall was about two times lower than the flux in the WB simulation. Figure 7*b* contains horizontal lines that have a higher platelet count than other areas in the domain. This effect is caused by the absence of RBCs that drives the platelets to the sides of the channel and by the periodic boundary conditions that were used. The platelets kept the same trajectory while looping from outlet to inlet.

The platelet residence time is shown on a logarithmic scale for both simulations in figure 7. In figure 7*a*,*b*, the platelets with the longest residence times were observed in the re-circulation areas in front of and behind the stenosis. However, note that we calculated the residence time as the time it takes a platelet to relocate its own diameter. The residence time of platelets near the wall was longer than in the centre of the domain for both simulations. The residence time at the wall in the stenotic area was 1–10 μs for WB and seemed to be lower for PRP, approximately 1–5 μs.

#### 3.1.2. Shear rates and shear stresses in van Rooij’s flow chamber

**Figure 8.**
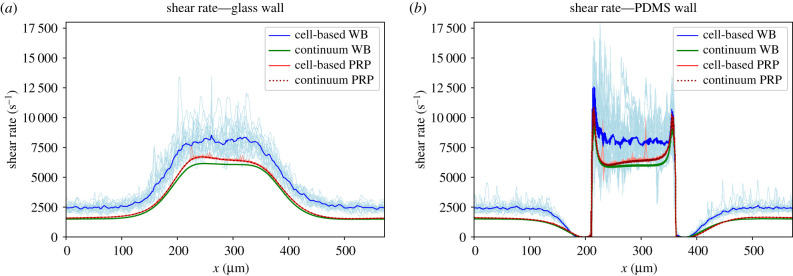
The *xy*-component of the shear rate measured at 1 μm from (*a*) the glass wall and (*b*) the PDMS wall in the cell-based and continuum blood flow simulations. The continuum WB simulation result is shown in green and the cell-resolved WB simulation results are shown in light blue for every 10 ms. The light red lines represent the cell-based PRP simulation for every 10 ms and the dotted dark red line represents the continuum PRP simulation. The average shear rate over time of the cell-based simulations is shown by the solid blue and the red lines, respectively, for WB and PRP.

**Figure 9.**
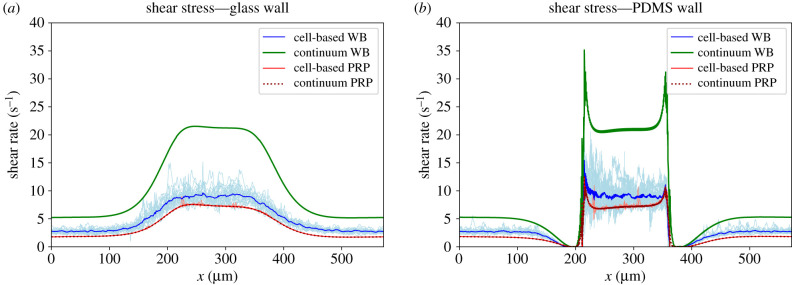
The *xy*-component of the stress measured at 1 μm from the (*a*) glass wall and (*b*) the PDMS wall in the cell-based and continuum blood flow simulations. The continuum WB simulation result is shown in green and the cell-resolved WB simulation results are shown in light blue for every 10 ms. The light red lines represent the cell-based PRP simulation for every 10 ms and the dotted dark red line represents the continuum PRP simulation. The averages of the cell-based simulations are shown by the solid blue and red lines, respectively, for WB and PRP.

The shear rates and shear stresses were measured 1 μm from the glass and PDMS wall in the cell-based simulations (figure 1*b*). The results obtained from the cell-based WB, continuum WB, cell-based PRP and continuum PRP simulation are shown in figures 8 and 9. The shear rate and shear stress were measured every 10 ms in the cell-based WB and PRP simulation and are shown as light blue and light red lines in those figures, respectively. The lines fluctuate for the shear rate between approximately 6000 and 12 000 s^−1^ on the glass wall and between 5000 and 15 000 s^−1^ on the PDMS wall. The average shear rate in the cell-based WB simulation was about 10–20% lower than the continuum WB simulation. The cell-based PRP simulation and its continuum simulation, however, showed the same shear rate distribution. Fluctuations could be neglected.

A lower shear stress was found in the WB cell-based simulation. The Newtonian fluid assumption for blood gave an overestimation of the shear stress close to the wall. The shear stress was increased approximately twice at both sides of the flow chamber. The shear stress of continuum PRP, however, was similar to the shear stress in the cell-based PRP simulation. In the electronic supplementary material, the shear stress averaged in the *z*-direction is shown for the cell-based and continuum WB simulation. These figures show that the shear stresses differ throughout the whole domain.

Near the PDMS wall large spikes were observed at both the upstream and downstream sides of the stenotic area. These are high shear rate gradients and are the result of the steep contraction and expansion angle of the stenosis in the flow chamber. In addition, the spikes might be artefacts because we show the *xy*-component of the shear rate/stress.

### 3.2. *In vitro* platelet aggregation experiments

**Figure 10.**
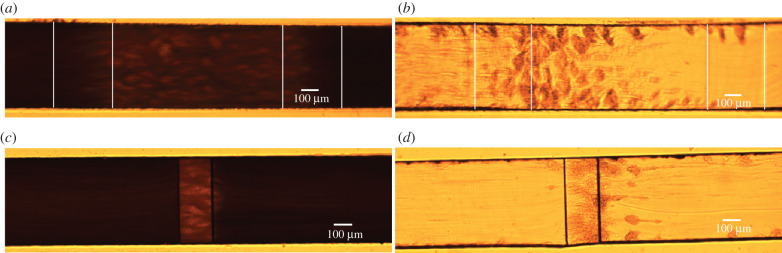
An example of an image at occlusion time for Casa’s flow chamber (*a*,*b*) and van Rooij’s flow chamber (*c*,*d*) perfused with WB and PRP. The vertical white lines in (*a*) and (*b*) indicate the contraction and expansion areas for Casa’s flow chamber. The stenotic section is located between the second and third white lines. (*a*) Casa WB, (*b*) Casa PRP, (*c*) van Rooij WB, (*d*) van Rooij PRP.

*In vitro* flow chamber experiments could provide more insight into the flow environment in which the platelet aggregates start to form. We investigated where the platelet aggregates start to form and compared this between Casa’s and van Rooij’s flow chambers. Additionally, we compared the starting location with the occlusion location in the flow chamber. Examples of platelet aggregates that formed in the two types of flow chambers perfused with WB and PRP (at occlusion time) are shown in figure 10. These frames were used in the analysis of the occlusion position in the flow chambers. Platelet aggregates formed in PRP were more visible than in WB owing to the absence of RBCs. In the electronic supplementary material, an example video is included for each type of experiment.

#### 3.2.1. Starting position of platelet aggregates in the experiments

**Figure 11.**
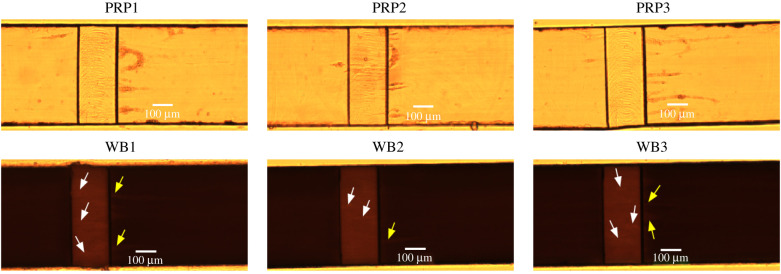
Examples of initial frames of platelet aggregate formation experiments in van Rooij’s flow chamber. The images were taken at the point in time when the first platelet aggregates became visible. In the top row, a selection of the PRP experiments is shown and on the bottom, a selection of the WB experiments. The white arrows point to small platelet aggregates on the surface of the stenotic section and the yellow arrows point to platelet strings that are attached to the back corner of the stenosis.

**Figure 12.**
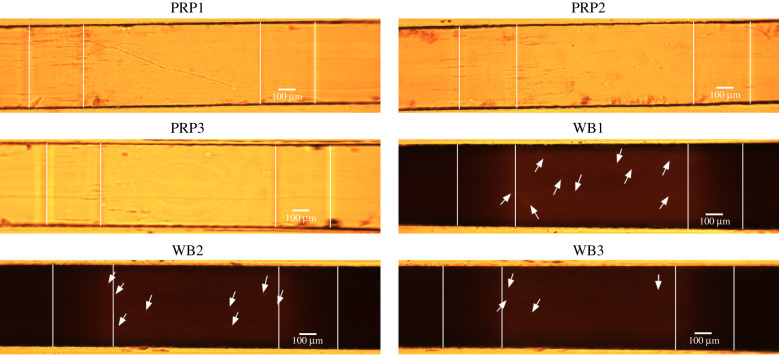
Examples of initial frames of platelet aggregate formation experiments in Casa’s flow chamber. The images were taken at the point in time when the first platelet aggregates became visible. In the top and the second row, a selection of the PRP experiments is shown. On the second and the bottom row, a selection of the WB experiments are shown. The white arrows point to small platelet aggregates on the surface of the stenotic section. The vertical white lines indicate the contraction and expansion areas of the flow chamber. The stenotic section is located between the second and third white lines.

In figures 11 and 12, a selection of images is shown for both types of flow chamber at the moment that platelet aggregates became visible. In van Rooij’s PRP experiments, we observed strings of platelets that formed from the downstream end of the stenotic area. The strings were straight or had a U-shape (see PRP1 and PRP2). Additionally, the first strings of platelet aggregates became visible in the stenotic part, which seemed to align with the flow. In the WB experiments, the strings of platelets were visible on the stenotic parts as lighter stripes (see white arrows). The strings from the downstream stenotic area were visible as lighter stripes as well (see yellow arrows in WB1 and WB2); however, they were not as visible as in PRP.

For Casa’s flow chamber, we observed a similar behaviour in the PRP experiments: strings of platelets that attached at the upstream side of the stenosis and in the contraction area. Additionally, we observed the formation of the platelet aggregates starting at the vertical sides of the flow chamber (*z* = 0 and *z* = width) most of the time. In the WB experiments, we could see the platelet aggregate stripes as well; however, they were less clear (see white arrows). In addition, platelet aggregates were observed in the middle and on the downstream side of the stenotic section, and not only at the upstream side of the stenosis in the WB experiments. It was more difficult to see if platelet aggregates were present in the contraction area than in the PRP experiments.

#### 3.2.2. Occlusion position of the platelet aggregate in the experiments

**Figure 13.**
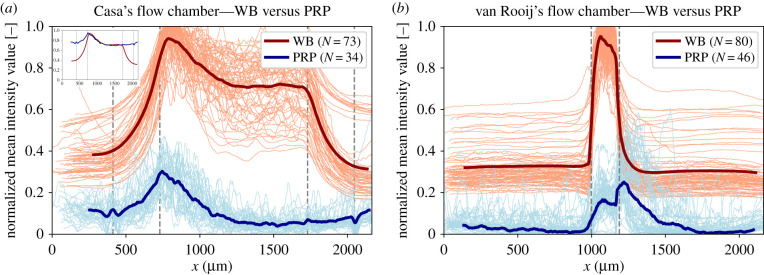
The normalized pixel intensities of (*a*) Casa’s flow chamber and (*b*) van Rooij’s flow chamber, measured from the occlusion frame, are shown by the light blue and the light red lines for the PRP and WB experiments, respectively. The thicker, darker lines show the average of WB (red) and PRP (blue). The vertical, dashed, grey lines define the contraction and expansion area in the middle of the stenotic area for Casa’s flow chamber and the stenotic area for van Rooij’s flow chamber. Flow was flowing from left to right. The inset image in (*a*) shows the averages of Casa’s flow chamber where the PRP curve is shifted on the *y*-axis.

The normalized mean pixel intensities of the WB and PRP experiments are shown in figure 13 for Casa’s flow chamber and van Rooij’s flow chamber. The light-coloured lines in the graphs present the individual experiments and the solid lines are the averages. What is interesting about these data is that the maximum pixel intensity, which indicates the part of the flow chamber where the number of platelets is the highest, was located in the stenotic section for both flow chambers. Additionally, a specific occlusion area could be distinguished from this graph for Casa’s flow chamber. The clot seems to form just after the contraction area in the stenotic section. Additionally, when the PRP average of the mean pixel intensities was shifted on the *y*-axis, the shape of the average WB and PRP curve matched very well (see figure 13, inset). From this, we assume that the occlusion area in Casa’s flow chamber is comparable in size and location for the WB and the PRP experiments. For van Rooij’s flow chamber, the WB experiments occluded in the stenotic section. However, an extra shoulder in the normalized pixel intensity curve of the PRP experiments was visible. We could presume that parts of the platelet aggregate also form in the channel behind the stenotic section.

The average distance from the start of the stenotic area to the platelet aggregates that occluded the channel was measured. This distance was obtained, for both flow chambers, from the corner on the upstream side of the stenotic area (see black arrows figure 2) to the point at which the normalized mean pixel intensity had reached its highest point. For Casa’s flow chamber, the average distance measured was 144 ± 26 μm for WB and 91 ± 50 μm for PRP, and for van Rooij’s flow chamber it was 85 ± 3.4 μm for WB and 285 ± 31 μm for PRP. The average distance measured in the PRP experiment with van Rooij’s flow chamber was larger than the stenotic area itself (150 μm). This was not the case for the WB experiment; however, it is possible that we could not see the platelet aggregate that formed in the flow chamber behind the stenotic section.

## 4. Discussion

In this study, the shear rate and shear stresses were investigated in van Rooij’s flow chamber using cell-based and continuum blood flow simulations. Furthermore, we studied if the cell-depleted layer in combination with high shear and high platelet flux can account for the distribution of platelet aggregates. We hypothesized that platelet aggregates might have different sizes and/or location in PRP from those in WB owing to differences in platelet fluxes.

We found a high-shear environment in the stenotic area of the flow chamber and a high platelet flux near the wall in the cellular WB simulation. This can be explained by the well-known phenomenon of margination of platelets caused by shear-induced collisions between platelets and RBCs [20]. In the cell-based PRP simulation, the RBCs were absent, which resulted in a lower flux of platelets close to the wall. Since platelets also experience lift force, albeit to a lesser degree than RBCs, they move further away from the wall. Therefore, a slightly higher platelet flux became visible in the layer closest to the wall in the cell-based PRP simulation. The platelet flux found next to the wall in WB is twice as high as that in the PRP cell-based simulation. This would suggest, from a transport physics viewpoint, neglecting the chemical part of the thrombus formation, that it would take twice as long for a platelet aggregate to form in the same flow chamber. An increase in occlusion time was found for PRP compared with WB experiments in this study (data not shown). This increase has been found by Mehrabadi [21] as well for PRP experiments in stenotic tubes owing to an increase in lag time. Additionally, two bands with a higher platelet concentration were observed at one-quarter and three-quarters of the height of the flow chamber in the cell-based WB simulation. It is unclear why this last division of platelets is present. It is assumed to be a volume exclusion effect, caused by the dense RBC core. These higher concentration levels seem to occur in cell-based simulations in a circular domain (3D) [20,22,23] as well as in two-dimensional domains [24]. The observation could be interesting for future investigations, e.g. margination studies in bifurcating vessels.

From the cell-based WB and PRP simulations, a platelet residence time of less than 10 μs was obtained. This means that the binding between a platelet and collagen or a platelet and vWF has to be faster than 10 μs. In the literature, it was found that the binding between vWF and platelet receptor GpIb*α* is the fastest bond in biology and is faster than 10 μs [25]. This makes it more likely that the mechanism in the microfluidic devices used in this study is based on the binding between vWF and platelets. Additionally, it is known that vWF attached (via collagen) to a wall uncoils at shear rates higher than 1000 s^−1^ [26]. The shear rates in the experiments, according to the simulations, were high enough to uncoil vWF and form platelet aggregates based on platelet binding to the vWF. Binding of an unactivated platelet directly to collagen via GPIV is not possible at shear rates higher than 1000 s^−1^ [27]. However, we are not sure if our platelets are unactivated from the start of our experiments. If platelets are activated a direct bond to collagen is possible via integrin *α*_2_*β*_1_ [27]. In our experiments, we clearly see strings of platelets (aggregates), which are probably formed by unfolded vWFs in the flow direction. Those unfolded vWFs have binding places available for platelets. The strings of platelets were observed under high shear by Ruggeri *et al.* [28] as well. This seems to imply that platelets are unactivated in our experiments and bind via vWF to the collagen coating. Additionally, this was confirmed by the discoid shapes found while counting the platelets with a haemocytometer.

Re-circulation areas in front of and behind the stenotic area were observed in the cell-based simulations. In these zones, platelets and RBCs were present from the initialization of the flow chamber with cells. Those re-circulation areas have been noted for the flow environment of stent struts [29]. The stenotic geometry has a comparable size to a stent strut. Therefore, this might indicate that similar re-circulation zones with trapped cells might exist in the vicinity of stent struts.

The experiments performed in this study gave insight into the occlusion location of the platelet aggregate in both flow chambers. From Casa’s experiments, we found that the platelet aggregates start to form preferably in the contraction area close to the stenotic section and at the upstream end of the stenotic section. The occlusion always occurred at the upstream part of the stenotic area. A reason for this could be that a high-shear environment is present in the stenotic area with a high platelet flux during the entire experiment. The fact that platelet aggregation in the contraction area of Casa’s flow chamber did not result in occlusion could be explained by a larger cross-sectional area. This might lead to faster occlusion in the stenotic section, because a smaller platelet aggregate and thus fewer platelets are needed. The WB experiments with van Rooij’s flow chamber showed final occlusion in the stenotic section. However, the platelet aggregate that occluded the flow chamber in the PRP experiments occupied a larger area, i.e. the stenotic area as well as the area just behind the stenosis. This area coverage could be due to the strings of platelets that started to form on the downstream corner of the stenosis.

From the platelet aggregation experiments in PRP, it was clear that RBCs are not needed to form a high-shear platelet aggregate. However, RBCs make the process faster (data not shown). This was also shown by Mehrabadi *et al.* [30]. Casa *et al.* [31] and Neeves *et al.* [32] stated that the start of thrombus growth is limited by the availability of (plasma) vWF and not by the availability of platelets. It is possible that the vWF concentration is higher at the walls owing to margination of vWF in its globular form. However, there is no evidence for the margination of vWF. Interestingly, we saw a platelet-free layer in the PRP simulation. This is probably caused by the lift force on the platelet induced by the wall. When we looked carefully at our WB simulation a small platelet-free layer was observed as well, next to the wall. This means that in both WB and PRP experiments there is a plasma layer present close to the wall which might be important for the binding of platelets and proteins. In our WB cell-based simulation with van Rooij’s flow chamber, a high shear rate, a high platelet flux and enough space were present for the formation of a platelet aggregate. Platelet aggregation occurs in PRP experiments, thus the cell-free layer is not a necessary condition and the computed platelet flux of approximately 1.5 platelets/s is still sufficient to start the formation of platelet aggregates. In addition, at the same flow rate a higher shear rate and shear stress were found in WB. This could be a reason that platelet aggregates were formed faster in WB. Additional experiments may be needed to indicate the platelet aggregates at their starting point and to study our possible indicators in more detail. These experiments should give a more detailed view of the binding of a few platelets to the wall of the flow chambers.

It was shown that the shear rate 1 μm from the wall is slightly underestimated and shear stress is overestimated when using the continuum WB simulation compared with the cell-based WB simulations. This confirms the results reported in a previous study [11]. A better approximation for the shear rate and stress could probably be reached when the non-Newtonian (shear-thinning) behaviour of blood is modelled, e.g. power-law fluid or Carreau fluid. This approach will be much faster than the cell-based simulations. However, it is plausible that these non-Newtonian models will not be able to show the fluctuations in shear rate and stress as we observed in the cell-based WB simulation (see light blue lines in figures 8 and 9). These fluctuations are probably caused by the presence of RBCs and platelets; however, this needs further investigation. No differences in shear rate and shear stress have been found between the cell-based PRP simulation and the continuum PRP simulation. This could be explained by the fact that the volume fraction of platelets was approximately 0.1%. For this volume fraction, the effect of intercellular collisions on the viscosity is negligible according to Einstein approximation [33]. Furthermore, slightly higher shear rate and shear stress were found at the contraction part of the flow chamber than at the stenotic part. A reason for this could be the sudden reduction in the height of the flow chamber that causes a steep increase in shear rate at the corner of the stenotic section. In addition, the sharp peaks in shear rate and stress at the PDMS wall could be artefacts caused by taking only the *xy*-component of the shear rate and stress into account.

Finally, some limitations of the *in vitro* experiments need to be discussed. First, porcine blood was used for the *in vitro* experiments that were performed in this study. The pig model has been used widely to mimic human haemostasis and thrombosis [2,34,35], but there are differences in RBC and platelet sizes and concentrations. However, despite these differences in porcine and human blood, thrombus formation seems to be similar [36,37]. Second, the shear rate and stress in the microfluidic device can slightly differ among experiments because the micro-machining technique has a relatively large standard error and causes surface roughness mainly in the contraction part of the flow chamber. However, Griffin *et al*. [2] found that the surface roughness has no influence on the location of the occlusive platelet aggregate in Casa’s flow chamber. Third, the use of a bright-field microscope has the limitation that it does not give any information on the height of the platelet aggregates, because the aggregation process is observed from the top (*xz*-plane). The intensity of the images was used to locate where the clot occluded the channel; however, the height could not be verified with microscope images. Therefore, a confocal microscope, where platelets are fluorescently labelled, could give more insights into initial platelet aggregation. Fourth, the number of RBCs was not counted. Therefore, the exact haematocrit of the experiment was unknown. However, the blood of normal pigs was used. The haematocrit of normal pigs does not vary much according to the literature [16]. Last, the blood that was used was heparinized, which can reduce thrombin generation and fibrin formation; however, we were not interested in those phenomena, but in platelet–vWF interactions.

## 5. Conclusion

Platelet aggregates and their flow environment were studied in van Rooij’s flow chamber. With our cell-based WB simulation, we confirm a slightly underestimated shear rate and overestimated shear stress with a continuum WB simulation. On the other hand, a continuum simulation will be a good approximation for the cellular PRP flow. In the *in vitro* experiments, platelet aggregation was different between WB and PRP, with different positions of the occlusive thrombi. Our cell-based simulation shows a higher flux and a higher shear rate in WB than in PRP. Therefore, cell-based modelling may help demonstrate that thrombus formation differences between WB and PRP may be, in part, due to margination effects on shear rate and platelet flux.

## Supplementary Material

Video 1: Cell-based simulation of whole blood

## Supplementary Material

Video 2: Whole blood experiment with Van Rooij's flow chamber

## Supplementary Material

Video 3: Platelet-rich plasma experiment with Van Rooij's flow chamber

## Supplementary Material

Video 4: Whole blood experiment with Casa's flow chamber

## Supplementary Material

Video 5: Platelet-rich plasma experiment with Casa's flow chamber

## Supplementary Material

Supporting_material_compbiomed_conference_paper3_clean.pdf

## Data accessibility

This article does not contain any additional data.
