# Supplementary material for: Haemodynamic flow conditions at the initiation of high-shear platelet aggregation: a combined in vitro and cellular in silico study
Source: Interface Focus. 2020 Dec 11;11(1):20190126. doi: 10.1098/rsfs.2019.0126 (PMC7739908; doi:10.1098/rsfs.2019.0126)
Supplement: Supporting_material_compbiomed_conference_paper3_clean.pdf [file rsfs20190126supp6.pdf]

Supporting Material

B.J.M. van Rooij[*,1], G. Závodszky[1], A.G. Hoekstra[1], and D.N. Ku[2]

1 Computational Science Lab, Informatics Institute, University of Amsterdam, Amsterdam, The Netherlands

2 Mechanical Engineering, Georgia Institute of Technology, Atlanta, United States of America

Mold

The mold of Van Rooij's microfluidic device is shown in Figure 1a and a manufactured microfluidic device is shown in Figure 1b. A 3D laser confocal microscope was used to measure the size of the channels on Van Rooij's microfluidic device. As discussed in the main text, the dimensions of two flow chambers were measured and their 3D images are shown in Figure 2.

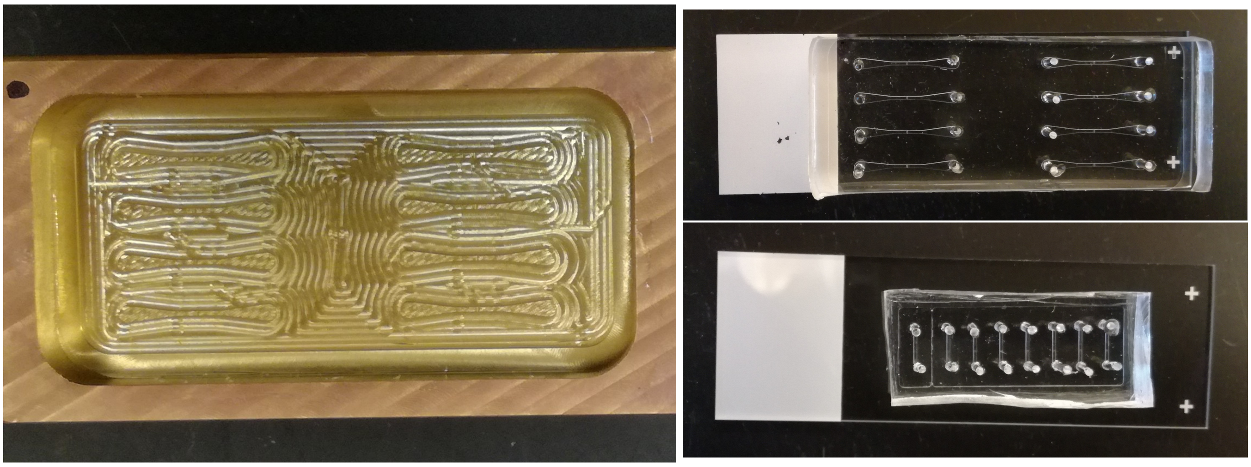

(a) (b)

Figure 1: (a) The micro-machining (brass) mold that is used to fabricated the PDMS-glass microfluidic devices; (b) the top image shows Van Rooij's microfluidic device and the bottom image showns Casa's microfluidic device.

Videos of cell-based simulations and experiments

Video 1: Cell-based simulation of whole blood with Q = $2.1 \cdot 10^{-9}$ m^3s^{-1}. In red, the red blood cells are shown and in yellow, the platelets. The video is slowed down to improve the visibility of the cells.
Video 2: Whole blood experiment with Van Rooij's (new) microfluidic device (Q = $2.4 \cdot 10^{-9}$ m^3s^{-1}). The edges of the stenotic section are marked by the vertical dashed lines. The video is playing five times faster than in reality.
Video 3: Platelet-rich plasma experiment with Van Rooij's (new) microfluidic device (Q = $2.4 \cdot 10^{-9}$ m^3s^{-1}). The

[*]B.J.M.vanRooij@uva.nl

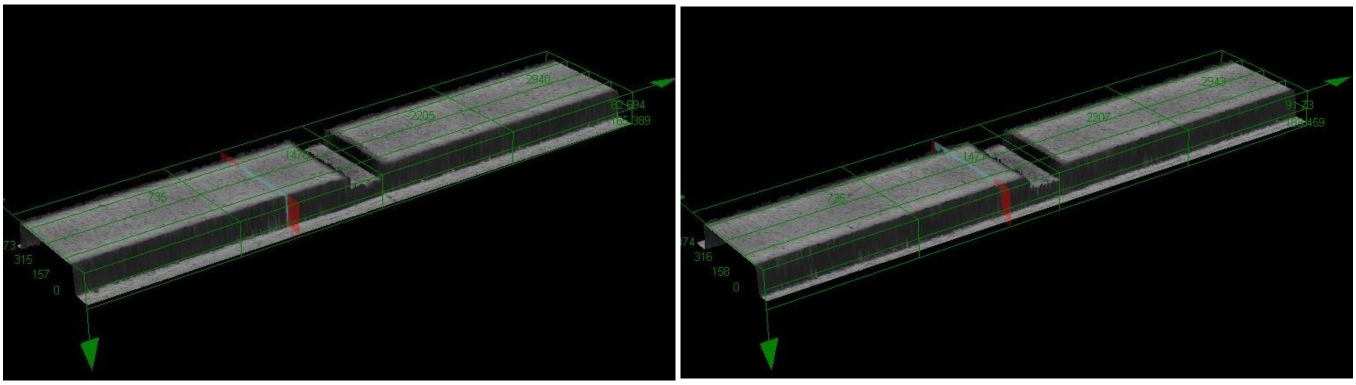

(a) (b)

Figure 2: A Van Rooij's (PDMS) device was scanned by a 3D industrial laser microscope to measure the size of the flow chamber and showed the precision of the micromachining technique. In those images the scanned surface of two flow chambers on the microfluidic device is shown.

edges of the stenotic section are marked by the vertical dashed lines. The video is playing ten times faster than in reality.
Video 4: Whole blood experiment with Casa's microfluidic device ($Q = 2.1 \cdot 10^{-9}$ m^3s^{-1}). The edges of the contraction and expansion sections are marked by the vertical dashed lines. The video is playing five times faster than in reality.
Video 5: Platelet-rich plasma experiment with Casa's microfluidic device ($Q = 4.9 \cdot 10^{-9}$ m^3s^{-1}). The edges of the contraction and expansion sections are marked by the vertical dashed lines. The video is playing ten times faster than in reality.

Shear stress in Van Rooij's flow chamber

The averaged xy-component of the shear stress for the continuous and cell-based WB simulation are shown in Figure 3. It is clearly visible that the shear stress in the cell-based simulation is very high at the sides and there is a large change in shear stress at the boundary of the cell-free layer (see inset Fig. 3a). For the continuum fluid case, the decrease in shear rate to the center of the flow chamber is linear (see inset Fig. 3b).

The capillary number of the cellular WB simulation is approximately 2.0 in the non-stenotic part of the flow chamber, and for the cellular PRP simulation it is approximately 1.6. The capillary number is calculated using the method of Krüger et al. [1].

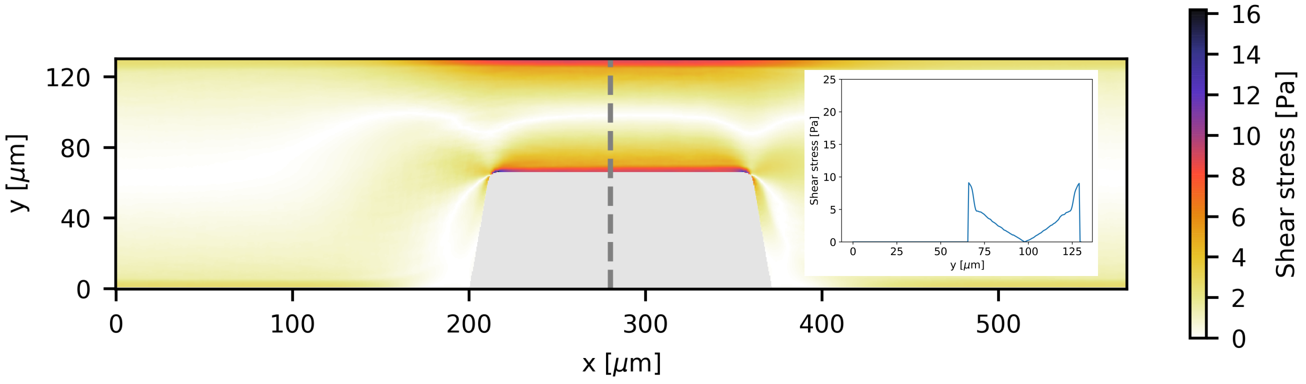

(a)

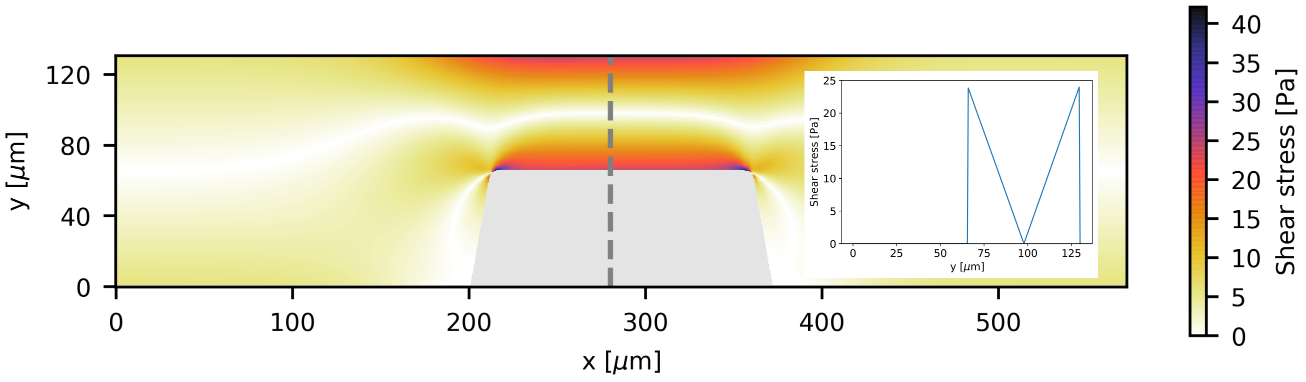

(b)

Figure 3: The averaged xy-component of the shear stress with a matched flow rate Q of $2.1 \cdot 10^{-9}$ m^3s^{-1} for (a) a cell-based WB simulation and (b) a continuum WB simulation of Van Rooij's flow chamber. The inset images show the averaged shear stress over the cross section at the location of the vertical dashed line.

References

[1] Timm Krüger. Effect of tube diameter and capillary number on platelet margination and near-wall dynamics. *Rheologica Acta*, 55(6):511–526, 2016. URL `https://doi.org/10.1007/s00397-015-0891-6`.
